# Intracellular amyloid beta expression leads to dysregulation of the mitogen-activated protein kinase and bone morphogenetic protein-2 signaling axis

**DOI:** 10.1371/journal.pone.0191696

**Published:** 2018-02-22

**Authors:** Eric Cruz, Sushil Kumar, Li Yuan, Jyothi Arikkath, Surinder K. Batra

**Affiliations:** 1 Department of Biochemistry and Molecular Biology, Fred and Pamela Buffett Cancer Center, University of Nebraska Medical Center, Omaha, Nebraska, United States of America; 2 Department of Pharmacology and Experimental Neuroscience, University of Nebraska Medical Center, Omaha, Nebraska, United States of America; 3 Developmental Neuroscience, Munroe-Meyer Institute, University of Nebraska Medical Center, Omaha, Nebraska, United States of America; Roswell Park Cancer Institute, UNITED STATES

## Abstract

Alzheimer’s disease (AD) is a neurodegenerative syndrome classically depicted by the parenchymal accumulation of extracellular amyloid beta plaques. However, recent findings suggest intraneuronal amyloid beta (iAβ_1–42_) accumulation precedes extracellular deposition. Furthermore, the pathologic increase in iAβ_1–42_ has been implicated in dysregulation of cellular mechanisms critically important in axonal transport. Owing to neuronal cell polarity, retrograde and anterograde axonal transport are essential trafficking mechanism necessary to convey membrane bound neurotransmitters, neurotrophins, and endosomes between soma and synaptic interfaces. Although iAβ_1–42_ disruption of axonal transport has been implicated in dysregulation of neuronal synaptic transmission, the role of iAβ_1–42_ and its influence on signal transduction involving the mitogen-activated protein kinase (MAPK) and morphogenetic signaling axis are unknown. Our biochemical characterization of intracellular amyloid beta accumulation on MAPK and morphogenetic signaling have revealed increased iAβ_1–42_ expression leads to significant reduction in ERK 1/2 phosphorylation and increased bone morphogenetic protein 2 dependent Smad 1/5/8 phosphorylation. Furthermore, rescue of iAβ_1–42_ mediated attenuation of MAPK signaling can be accomplished with the small molecule PLX4032 as a downstream enhancer of the MAPK pathway. Consequently, our observations regarding the dysregulation of these gatekeepers of neuronal viability may have important implications in understanding the iAβ_1–42_ mediated effects observed in AD.

## Introduction

Alzheimer’s disease (AD) is the most frequently occurring neurodegenerative disorder, affecting over 5 million men and women in the United States [1]. Given the extraordinary prevalence of this illness and the ever-increasing socioeconomic toll, investigation of novel strategies to combat AD is imperative. Though the molecular etiological determinants of AD has extensively been investigated, the mechanism in which these changes elicit neurodegeneration remains poorly resolved. While numerous factors may contribute to the neuropathological transformation associated with AD, the pathognomonic accumulation of amyloid beta resides in the forefront as a diagnostic tool and therapeutic target [2].

Formation of the amyloid beta peptide results from metabolism of the amyloid precursor protein (APP), wherein, cleavage by β-secretase and γ-secretase, yield an amyloidogenic peptide fragment. This peptide is of varying size with the 42 amino acid isoform considered the most amyloidogenic [3]. Subsequently, the assembly of extracellular amyloid beta (eAβ) into oligomers and fibrils contribute to the hallmark parenchymal deposition and pathological activity. However, while eAβ has historically garnered the greatest attention, the intracellular localization of amyloid beta (iAβ) is receiving increasing consideration for its pathophysiological contributions to AD [4].

Indeed, support for realignment of the amyloid central dogma is evident from *in vivo* observations employing genetically engineered mouse models of AD and post-mortem examination of patients with heritable early onset AD (EOAD). Remarkably, these studies have revealed iAβ expression to precede extracellular plaque deposition with the intracellular colocalization observed within endosomes, multivessicular bodies, lysosomes, mitochondria, endoplasmic reticulum, and cytosol [3, 5, 6]. Furthermore, the cognitive deficits, behavioral changes, and axonal degeneration associated Alzheimer’s type pathologies are more closely linked to intraneuronal accumulation of Aβ than that of extracellular plaque deposition [7]. At the cellular and molecular level, intraneuronal Aβ accumulation has been shown to impair aspects of axonal transport and synaptic transmission thereby suggesting a role in the cognitive impairment associated with AD [8–10]. However, in addition to the impact on synaptic transmission, iAβ may have an equally important, yet unexplored, influence on signal transduction. Although these processes are functionally unique, they have analogous dependence on axonal transport for regulation of their stability and activity [11, 12]. To understand how iAβ may influence signal transduction, we have focused our investigation on mitogen-activated protein kinase (MAPK) and morphogenetic signaling activity. Neurotrophin mediated signaling (i.e. BDNF, NGF, etc.) has long been regarded for its ability to stimulate nerve growth, preserve axonal integrity, and maintain neuronal viability [13, 14]. In contrast, bone morphogenetic protein signaling (i.e. BMP-2, BMP-4, etc.) are key regulators of programmed cell death during interdigital apoptosis, skeletal remodeling, and neural crest reorganization [15, 16]. However, in sensory neurons, the morphogenetic signaling mediated by BMPs, phosphorylate Smad1 leading to Erk1/2 transcriptional upregulation and enhance neurotrophic response. Therefore, morphogenetic and neurotrophic signaling is tightly regulated during synaptic connectivity, suggesting the context dependent crosstalk between these signaling pathways [17]. Furthermore, the divergent activity of these pathways are pivotal in axonal pruning and synaptic regression which form the foundation for a signaling axis that is essential for maintaining neuronal transduction and establishment of the adult neural network [18–20].

To study the effects of iAβ on these critical pathways, we have employed a lentivirus iAβ overexpression system. Interestingly, upon iAβ expression, we have observed dramatic alterations to the MAPK and morphogenetic signaling pathways. Indeed, in the context of iAβ expression, significantly diminished ERK1/2 phosphorylation was observed. In contrast, induction of BMP-2 dependent receptor Smad 1/5/8 phosphorylation was significantly elevated in iAβ expressing cells. Combined, the inversely proportional disruption of these signaling programs may alter essential pathways necessary for maintaining neurotrophic and morphogenetic signaling flux. Thus, our elucidation of the fundamental molecular and biological features associated with iAβ accumulation may have critically important implications in the neurodegeneration observed in clinical AD.

## Materials and methods

### Plasmids and cloning strategy

Cloning of the iAβ_1–42_ in the lentivirus expression vector was accomplished by RT-PCR amplification of HEK293 [ATCC, Manassas, VA (CRL-1573)] derived cDNA corresponding to the Aβ_1–42_ CDS followed by cloning into PCR2.1 using the TOPO TA cloning Kit (Invitrogen, Carlsbad, CA). Next, the insert was subcloned into the pLenti-EF1a-GFP-2A-Puro vector. All constructs were sequenced by the UNMC High-Throughput DNA Sequencing and Genotyping Core Facility to ensure proper composition. Generation of the recombinant lentivirus used for HEK293 transduction was accomplished by plasmid transfection of the shuttle vector into Lenti-X 293T packaging cells (Takara Bio USA, Inc., Mountain View, CA). The viral supernatant was collected and further concentrated by use of the Lenti-X Concentrator solution (Clontech, Mountain View, CA). The high titer generation of the recombinant lentivirus was performed by the Viral Vector Core Facility at the University of Iowa, Carver College of Medicine (Lot #H677 and Lot #H680). To increase the infection efficiency and limit toxicity, magnetic-bead based transduction was performed using the Lenti-X Accelerator solution (Takara Bio USA, Inc., Mountain View, CA). The Lenti-III-EF1alpha-GFP control virus (Applied Biomedical Materials) was employed for biological comparisons. The pLenti-EF1a-GFP-2A-Puro vector was kindly provided by the laboratory of Dr. Keith R. Johnson. The primers, iAβ_F:5’*GGATCCGCCACCATGGAACAAAAACTCATCTCAGAAGAGGATCT GGATGCAGAATTCCGACATGACTC-3’*, *and* iAβ_R *5’-AGATCTGACGCTATGACAA CACCGCCCACCATGAGTCCAATG-3’* were employed for the various expression vector constructs.

### Primary rat hippocampal and cortical neurons and cell lines

The primary rat hippocampal and cortical neurons were isolated from embryonic day 18 (E18) rat pups wherein the hippocampi or cortices were dissected, triturated, trypsinized, and seeded to poly-L-lysine coated plates/coverslips. Briefly, pregnant rats were obtained from a commercial source (Sprague-dawley, Charles River Laboratories, CA) and housed at the UNMC animal facility with water and food provided *ad libitum*. The pregnant rat containing 7 to 12 embryos was anesthetized using Isoflurane before cervical dislocation to minimize suffering and distress. During a single culture preparation, neurons from all embryos are pooled and plated out and the numbers indicated in the manuscript are used for each experiment. Each N represents data obtained from embryos from one pregnant rat. Hippocampal and cortical neurons were maintained in Neruobasal^®^ media containing B27 supplement. Cells were matured and analyzed after 21 days *in vitro* (DIV21). Hippocampal cells were seeded at an initial density of 1.875x10^5^/ cm^2^ and cortical cells seeded at 5.0x10^5^/ cm^2^ [21]. Care for animals and primary neuronal isolation procedure was approved and performed in accordance with University of Nebraska Medical Center institutional care and use committee. The primary rat hippocampal neuronal cells at DIV17 were transduced with the lentivirus construct. In addition, HEK293 cell line obtained from ATCC, Manassas, VA (CRL-1573) was used for the biochemical analysis after lentivirus transduction.

### Western blotting

Cells were lysed with RIPA buffer supplemented with cOmplete protease inhibitor cocktail (Roche, Indianapolis, IN), 8 mM sodium fluoride, 0.5 mM phenylmethylsulfonyl fluoride (PMFS), 1 mM sodium orthovanadate, and 2.5 mM sodium pyrophosphate. Protein was resolved through SDS-PAGE under denaturing conditions, transferred to polyvinylidene difluoride (PVDF) membrane, and blocked in 5% BSA containing PBS or TBS (Cold Spring Harbor Protocols, 2017). Subsequent incubation with the indicated antibody was done overnight at 4°C. Incubation with HRP-conjugated secondary in PBS or TBS containing 3% BSA was performed for 1 h and protein was detected using ECL chemiluminescence methods (Pierce ThermoScientific, Grand Island, NY). Experiments were performed in biological replicates. Densitometry analysis and 2-D gel transformation was performed using the LI-COR Image Studio Lite software (LI-COR).

### Reagents and antibodies

Reagents were obtained from the following sources: BDNF from PeproTech, Rocky Hill, NJ (450–02); rhBMP-2 from Life Technologies Gibco^®^. PLX4032 (Vemurafenib) from Selleckchem; EGF from ProSpec; NGF (mouse NGF 2.5S) from Thermo Fisher Scientific, Waltham, MA. Antibodies were obtained from the following sources: Alexa Fluor 568 conjugated secondary from Life Technologies; β-Actin from Sigma-Aldrich; HRP-Conjugated mouse (31430) and rabbit (31460) secondary from Pierce Thermo Scientific; total-Smad 1/5/8 (sc-6031), total-EGFR (SC03), and phospho-EGFR (S1046) (sc-101665) from Santa Cruz Biotechnology, Dallas, TX; Myc, phospho-Smad 1/5/8 (13820), phospho-EGFR (Y1068), phospho-Raf (Ser259) (9421), total-ERK1/2 (9102), and phospho-ERK 1/2 (9101) from Cell Signaling Technology, Danvers, MA.

### Phospho-flow cytometry

Flow cytometry was conducted on the BD LSR II instrument. Subsequent analysis was performed using BD FACSDiva 8.0 software for determination of median fluorescence intensities (MFI). Briefly, stably expressing GFP positive VC or iAβ expressing HEK293 cells were treated with EGF or BMP-2 for 1 h, fixed with 4% paraformaldehyde for 10 min, and permeabilized with ice cold methanol for 3 min. Following 1 h incubation with 1° antibody, and subsequent 1 h incubation with Alexa Fluor 568 conjugated 2° antibody, the cells were analyzed by flow cytometry. The GFP-positive population was then assessed for phospho-ERK 1/2, phospho-EGFR, or phospho-Smad 1/5/8. Phospho-flow cytometry performed at UNMC Flow Cytometry Research Facility for the generation and analysis of this data.

### Amyloid beta peptide oligomerization

The synthetic monomeric β-Amyloid_1-42_ peptide (*DAEFRHDSGYEVHHQKLVFFAEDVGSNKGAIIGLMVGGVVIA*), purchased from AnaSpec, Fremont, CA was prepared by dissolving the lyophilized peptide in hexafluoroisoproponol, dried under nitrogen steam, and then resuspended in DMSO to a concentration of 10 mM. For subsequent preparation of oligomeric forms of the peptide, the 10 mM stock was dissolved in DMEM at 25 μM concentration and incubated at 4°C for 24 h. Alternatively, for fibrillary β-Amyloid formation, the 10 mM stock was resuspended in PBS and incubated at 37°C for 24 h while being rotated. Cells, were then pretreated with 2 μM of the soluble or fibrillary extracellular Aβ_1–42_ 24 h prior to any subsequent treatment or analysis [22, 23].

### Statistical analysis

Statistical analysis of biological replicates was performed by two-sided, unpaired student’s t-test wherein p < 0.05 was taken to be statistically significant. In instances where outliers were considered, Dixon’s outlier analysis was performed on the data when indicated with samples excluded if p < 0.05. Generation of graphical data was accomplished with SigmaPlot 11.2. Using MedCalc, a Kruskal-Wallis test (H-test) was performed to assess the statistical significance of the differences in ERK 1/2 phosphorylation observed upon transduction with either VC or iAβ and subsequent treatment. Pairwise comparison of the subgroups was performed and shown in Tables 1–6.

**Table 1:** Kruskal-Wallace test (p < 0.05) with post-hoc pairwise analysis of ERK 1/2 phosphorylation in VC and iAβ transduced HEK293 cells treated in presence or absence of eAβ_1–42._

Factor	n	Average Rank	Different (p < 0.05) From Factor n
(1) iAβ_DMSO	3	5.00	(2)(3)(4)
(2) iAβ + M/O/F	3	2.00	(1)(3)(4)
(3) VC_DMSO	3	10.00	(1)(2)
(4) VC + M/O/F	3	9.00	(1)(2)

**Table 2:** Kruskal-Wallace test (p < 0.05) with post-hoc pairwise analysis of HPC and CTX ERK 1/2 phosphorylation following NGF treatment with and without PLX4032.

Factor	n	Average Rank	Different (p < 0.05) from factor n
1) CTX_DMSO	3	6.33	(4)(5)(6)
(2) CTX_NGF	3	3.33	(4)(5)(6)
(3) CTX_PLX NGF	3	5.33	(4)(5)(6)
(4) HPC_DMSO	3	12	(1)(2)(3)(6)
(5) HPC_NGF	3	13	(1)(2)(3)
(6) HPC_PLX_NGF	3	17	(1)(2)(3)(4)

**Table 3:** Kruskal-Wallace test (p < 0.05) with post-hoc pairwise analysis of HPC and CTX ERK 1/2 phosphorylation following BDNF, EGF, and/or PLX4032 treatment.

Factor	n	Average Rank	Different (p < 0.05) from factor n
(1) CTX_BDNF	3	8.33	(7)(8)(9)(10)(11)(12)
(2) CTX_DMSO	3	8.33	(7)(8)(9)(10)(11)(12)
(3) CTX_EGF	3	9	(7)(9)(10)(11)(12)
(4) CTX_PLX	3	5.67	(6)(7)(8)(9)(10)(11)(12)
(5) CTX_PLX_BDN	3	16.33	(7)(9)(11)
(6) CTX_PLX_EGF	3	18.67	(4)(7)
(7) HPC_BDNF	3	31	(1)(2)(3)(4)(5)(6)
(8) HPC_DMSO	3	20.67	(1)(2)(4)
(9) HPC_EGF	3	28.67	(1)(2)(3)(4)(5)
(10) HPC_PLX	3	22	(1)(2)(3)(4)
(11) HPC_PLX_BDN	3	29	(1)(2)(3)(4)(5)
(12) HPC_PLX_EGF	3	24.33	(1)(2)(3)(4)

**Table 4:** Kruskal-Wallace test (p < 0.05) with post-hoc pairwise analysis of Smad 1/5/8 phosphorylation following BMP-2 and Dorsomorphin treatment of HPC and CTX cells.

Factor	n	Average Rank	Different (p < 0.05) from factor n
(1) CTX_1X BMP	3	4.33	(4)(5)
(2) CTX_2X BMP	3	6.33	(4)(5)
(3) CTX_2X BMP_DORS	3	5.67	(4)(5)
(4) HPC_1X BMP	3	15	(1)(2)(3)
(5) HPC_2X BMP	3	15.67	(1)(2)(3)
(6) HPC_2X BMP_DORS	3	10	

**Table 5:** Kruskal-Wallace test (p < 0.05) with post-hoc pairwise analysis of ERK 1/2 phosphorylation following BDNF and PLX4032 treatment of VC or iAβ transduced HPC cells.

Factor	n	Average Rank	Different (p < 0.05) from factor n
(1) IAB_BDNF	3	8	(2)(3)(4)(6)
(2) IAB_DMSO	3	3	(1)(3)(4)(6)
(3) IAB_PLX_BDNF	3	13.33	(1)(2)(5)
(4) VC_BDNF	3	14.33	(1)(2)(5)
(5) VC_DMSO	3	4	(3)(4)(6)
(6) VC_PLX_BDNF	3	14.33	(1)(2)(5)

**Table 6:** Kruskal-Wallace test (p < 0.05) with post-hoc pairwise analysis of phospho-Smad 1/5/8 following rh-BMP2 and/or Dorsomorphin treatment of VC or iAβ transduced HPC cells.

Factor	n	Average Rank	Different (P<0.05) from factor n
(1) HPC_IAB_BMP2	3	17	(2)(3)(5)(6)
(2) HPC_IAB_DMSO	3	4.5	(1)(4)
(3) HPC_IAB_DORSO_BMP2	3	10.17	(1)
(4) HPC_VC_BMP2	3	11.33	(2)(5)
(5) HPC_VC_DMSO	3	4.5	(1)(4)
(6) HPC_VC_DORSO_BMP2	3	9.5	(1)

## Results

### iAβ expression leads to decreased total-EGFR and phospho-EGFR abundance following EGF stimulation

**Fig 1:**
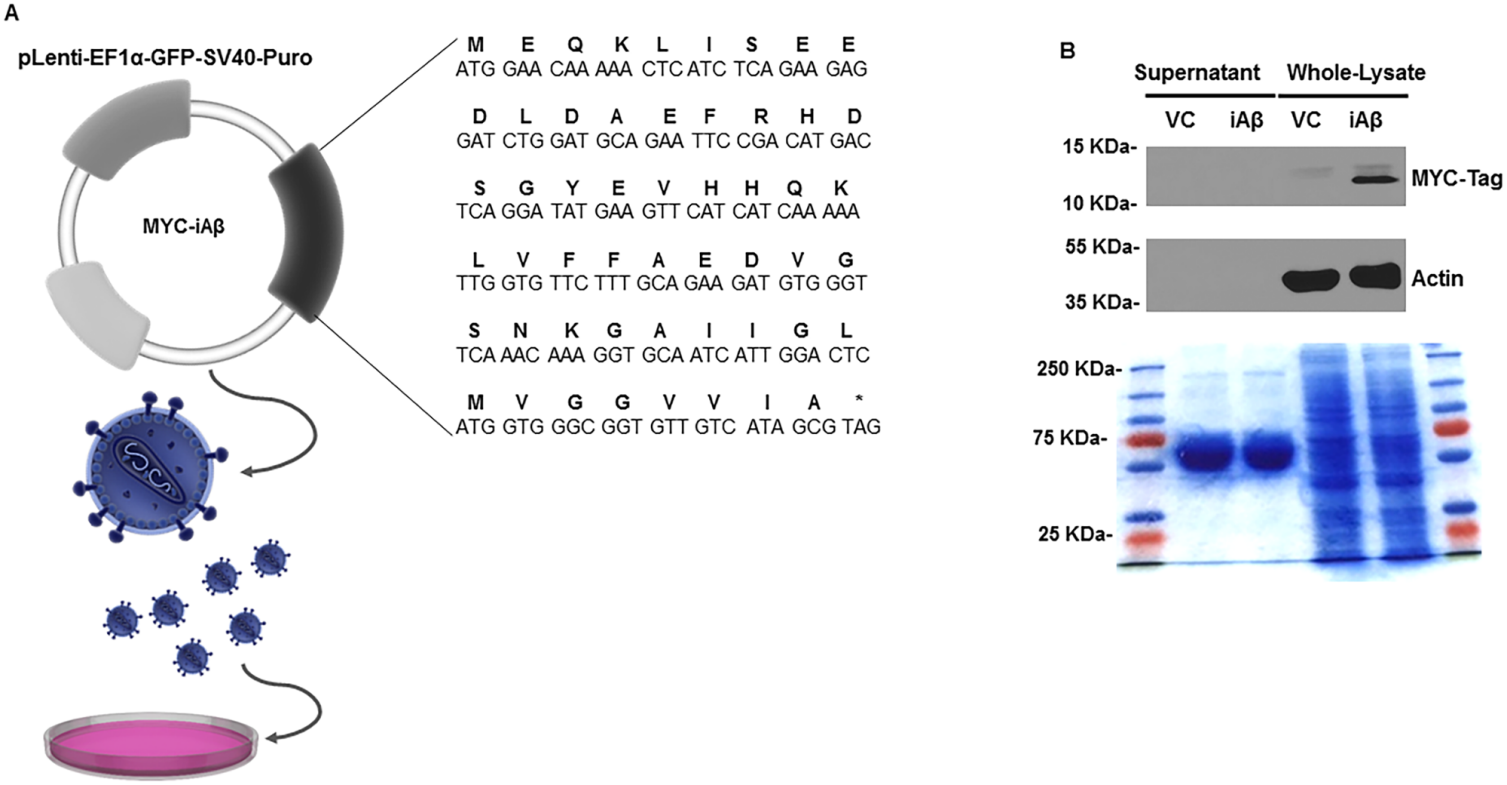
Validation of expression and localization of iAβ peptide. **(A)** Schematic of the primary nucleotide and corresponding amino acid sequence of the MYC-Tagged iAβ used for cloning into the pLenti-EF1α-GFP-SV40-Puro lentiviral shuttle vector (Images adapted from Library of Science and Medical Illustrations CC BY-NC-SA 4.0). **(B)** Immunoblot of the concentrated supernatant and the whole cell-lysates of VC or iAβ transduced HEK293 cells for MYC-Tagged iAβ expression. Expression of the MYC epitope tag is observed in the whole cell-lysates of samples corresponding iAβ transduced cells only. Further, MYC-tagged iAβ is present in samples obtained from the whole cell-lysates and absent in samples obtained from the cultured supernatant confirming its intracellular retention. Coomassie brilliant blue stained, 12% SDS PAGE gel, of both concentrated supernatant and total cell lysates in VC and iAβ transduced cells demonstrates equal loading among samples.

Persistent neurotrophic support, facilitated by ligand-receptor mediated activation of the MAPK pathway (RAS-RAF-MEK-ERK), is critical to preserving neuronal cell viability [24]. To assess the extent to which iAβ expression affects receptor stability and activity, we have generated an intracellular, N-terminally MYC-tagged Aβ_1–42_ lentivirus expression system (Fig 1A). Examination of both the cultured supernatant and the whole-cell lysates of pLenti-EF1α- GFP-SV40-Puro vector alone (VC) or pLenti-EF1α- GFP-SV40-Puro Myc-iAβ_1–42_ (iAβ) transduced HEK293 by immunoblotting was performed to confirm iAβ peptide expression and localization. As shown, detection of the MYC-tagged peptide could be observed in lanes corresponding to the iAβ-transduced samples, but not in the VC (Fig 1B). Further, we observed iAβ peptide expression in the whole-cell lysates, but not in the concentrated supernatant thereby confirming an intracellularly restricted localization.

**Fig 2:**
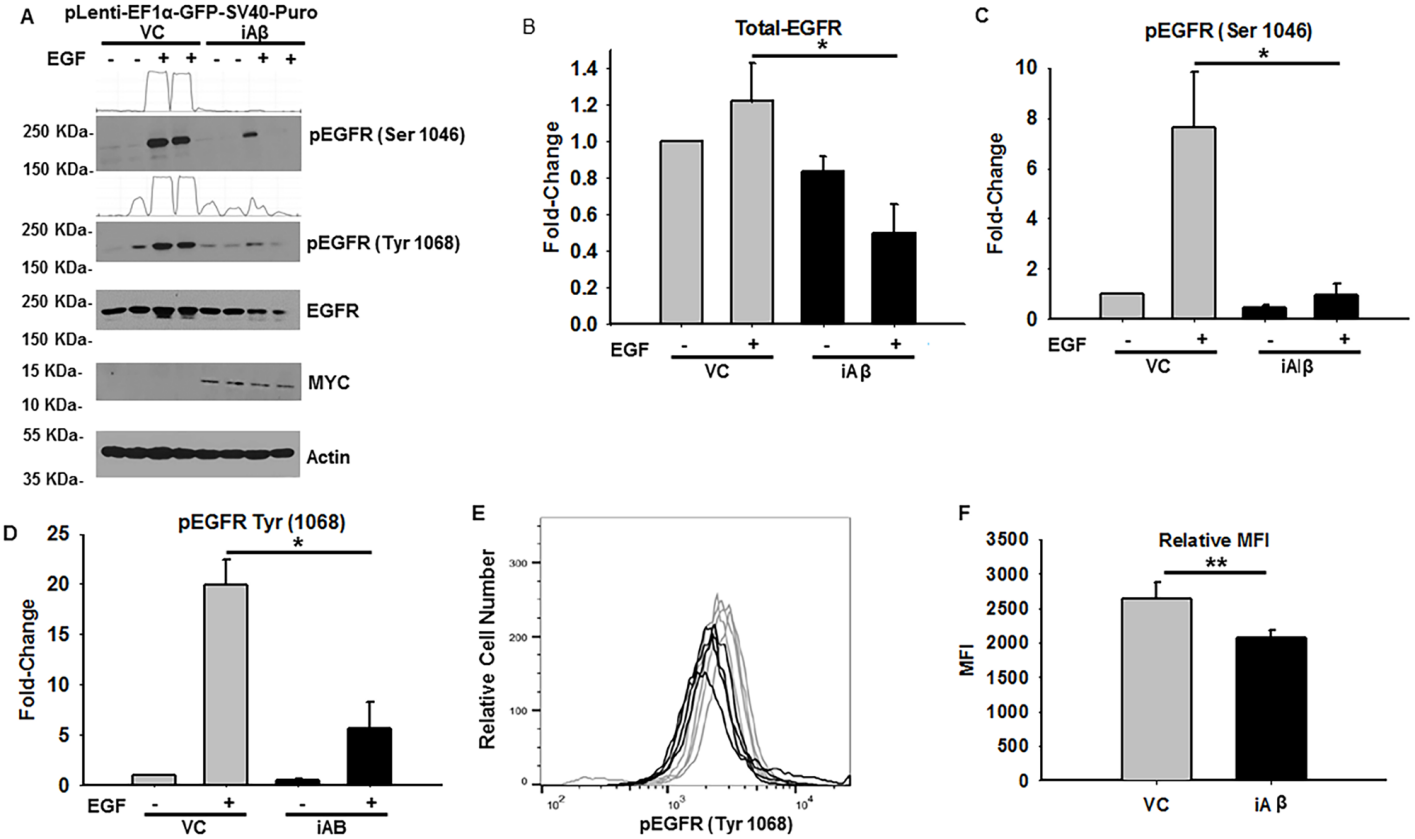
iAβ expression leads to relative reduction in total-EGFR and phospho-EGFR abundance following EGF stimulation. **(A)** Immunoblot of phospho-EGFR (Ser 1046 & 1068), total-EGFR, and MYC-Tag in VC or iAβ transduced HEK293 cells following EGF stimulation. MYC-Tagged protein expression is seen in lanes corresponding to iAβ transduced cells only. Reduction in total-EGFR and phospho-EGFR intensities are apparent in EGF treated iAβ cells relative to VC. Two-dimensional gel transformation of phospho-EGFR intensities performed and shown as histogram. Beta-actin used as loading control. **(B)** Plot of the relative densitometry analysis of total-EGFR expression demonstrates a statistically significant (*p < 0.05) decrease in EGFR abundance in iAβ expressing cells upon EGF stimulation. **(C & D)** Analysis of phospho-EGFR (Ser 1046 and Tyr 1068) depicts the statistically significant (*p < 0.05) reduction in phospho-EGFR intensities in EGF treated iAβ cells relative to VC. Experiment performed in biological replicates with data shown as mean ± SE (n = 3) and statistical significance. **(E)** Histogram of phospho-flow cytometry analysis of EGF treated VC (gray) or iAβ (black) transduced HEK293 cells. Shift in the MFI of iAβ transduced cells relative to the VC reflects a decrease in the phospho-EGFR (Tyr 1068) abundance. **(F)** Graphical depiction of replicate analysis shown as mean ± SE (n = 3) of the MFI with a statistically significant (**p < 0.01) 1.26-fold decrease in MFI.

Subsequent examination of the basic mechanistic and functional impact that iAβ expression has on EGFR status was measured in stably selected HEK293 cells transduced with VC or iAβ. We next performed immunoblotting for total-EGFR and phospho-EGFR following treatment with 50 ng/mL of EGF for 1 h. While there were no observable differences under unstimulated conditions, a relative decrease in both total-EGFR and phospho-EGFR was seen upon EGF stimulation (Fig 2A). The corresponding intensities, relative to VC samples treated with DMSO, were quantified and plotted. Indeed, a statistically significant (p < 0.05) 2.47-fold decrease in total-EGFR levels was seen following EGF stimulation (Fig 2B). Furthermore, a statistically significant (p < 0.05) decrease of 8.0-fold and 3.5-fold was observed in phospho-EGFR (Ser 1046) and phospho-EGFR (Tyr 1068) in the context of iAβ expression (Fig 2C and 2D). As a secondary measure of relative EGFR phosphorylation, phospho-flow cytometry was performed and demonstrated a comparable and statistically significant (p < 0.01) 1.27-fold decrease in phospho-EGFR (Tyr 1068) abundance (Fig 2E and 2F). Combined, our biochemical assessment of EGFR status has revealed an iAβ dependent decrease in both EGFR stability and phosphorylation that may consequently disrupt downstream signal intensity and duration.

### iAβ expression results in impaired MAPK activation

**Fig 3:**
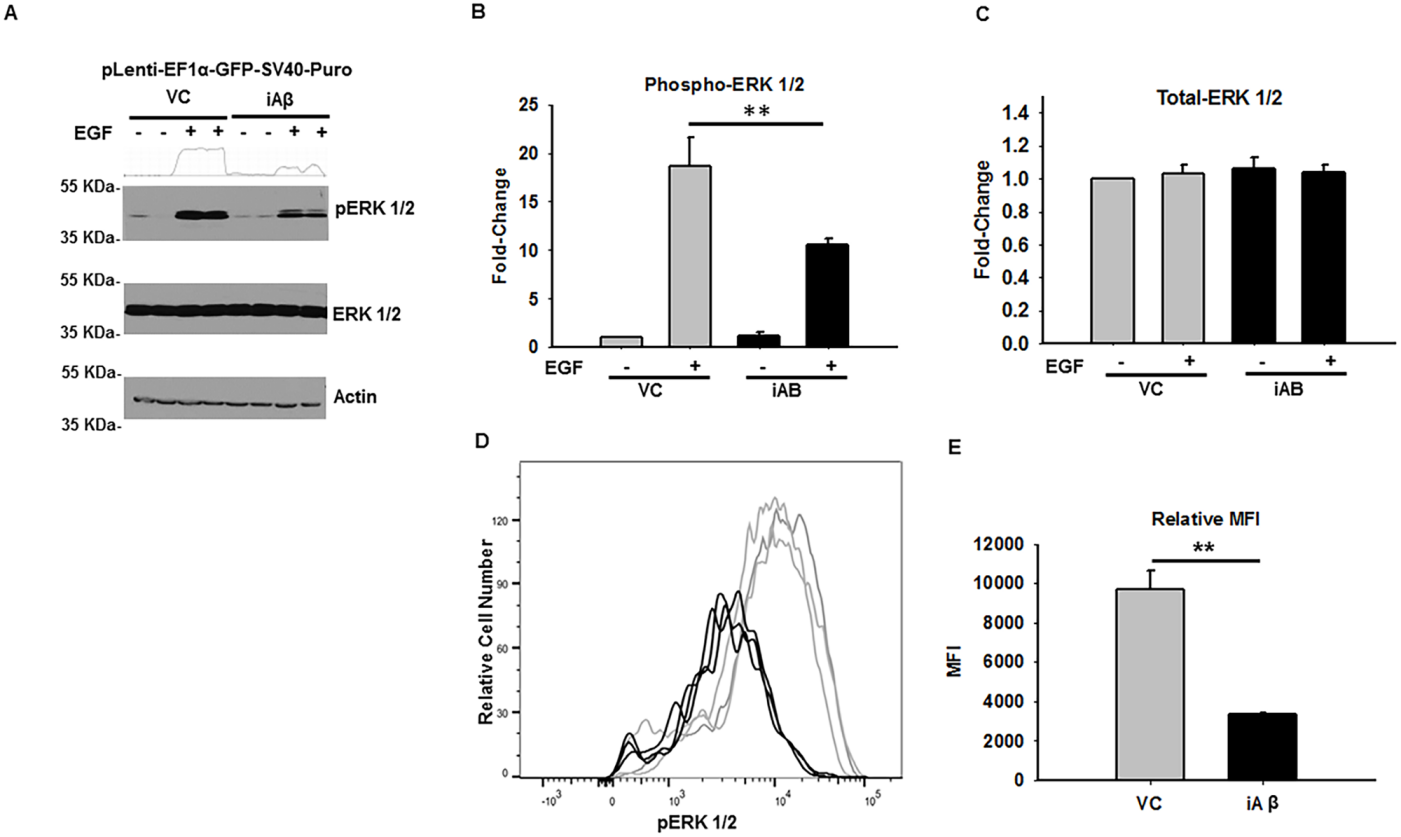
iAβ expression leads to diminished EGF-dependent ERK1/2 phosphorylation. **(A)** Immunoblot of phospho-ERK 1/2 and total-ERK 1/2 in VC or iAβ transduced HEK293 cells following EGF stimulation. While total-ERK 1/2 remains unaltered, decreased EGF dependent phospho-ERK 1/2 levels observed in iAβ-transduced cells relative to VC. Two-dimensional gel transformation of phospho-ERK 1/2 intensities performed and shown as histogram. Beta-actin used as loading control. **(B)** Plot of the relative densitometry analysis in biological replicates depicting a relative decrease in phospho-ERK 1/2 levels with data shown as mean ± SE (n = 3) with statistical significance (**p < 0.01). **(C)** Plot of the relative total-ERK 1/2 expression demonstrating unaltered expression among groups. **(D)** Histogram of phospho-flow cytometry analysis of EGF treated VC (gray) or iAβ (black) transduced HEK293 cells. A shift in the MFI of iAβ relative to VC reflecting a decrease in EGF dependent ERK 1/2 phosphorylation. **(E)** Graphical representation of replicate analysis shown as mean ± SE (n = 3) of the MFI with statistically significant (**p < 0.01) 2.63-fold decrease in MFI.

Although EGFR abundance and phosphorylation was found to be decreased in the context of iAβ expression, we sought to determine if downstream ERK 1/2 phosphorylation was similarly impaired. Thus, stably selected VC and iAβ transduced HEK293 cells were treated with vehicle or 50 ng/mL of EGF for 1 h and ERK 1/2 phosphorylation was measured by immunoblotting. Interestingly, while no changes in total-ERK 1/2 were observed, a substantial decrease in phospho-ERK 1/2 intensity was seen in iAβ expressing cells relative to VC (Fig 3A). Indeed, plots of the densitometry analysis, relative to VC samples treated with DMSO, reveals similar expression of total-ERK 1/2 is present in both VC and iAβ samples. In contrast, a striking difference in ERK 1/2 phosphorylation was detected upon EGF stimulation. Indeed, as compared with VC, the iAβ transduced cells demonstrate a statistically significant (p < 0.01) 2.91-fold decrease in ERK 1/2 phosphorylation (Fig 3B and 3C).

To further examine the EGF responsiveness in the context of iAβ expression, phospho-flow cytometry analysis was performed on VC and iAβ transduced HEK293 cells (Fig 3D). Analysis of cells treatment with 50 ng/mL of EGF for 1 h, by indirect flow cytometry revealed a comparable and statistically significant (p < 0.01) 2.63-fold decrease in ERK 1/2 phosphorylation (Fig 3E). Collectively, these observations demonstrate a corresponding decrease in ERK 1/2 phosphorylation resulting from the iAβ dependent dysregulation of EGFR stability and phosphorylation.

### iAβ leads to relative increase in BMP-2 dependent Smad 1/5/8 phosphorylation

**Fig 4:**
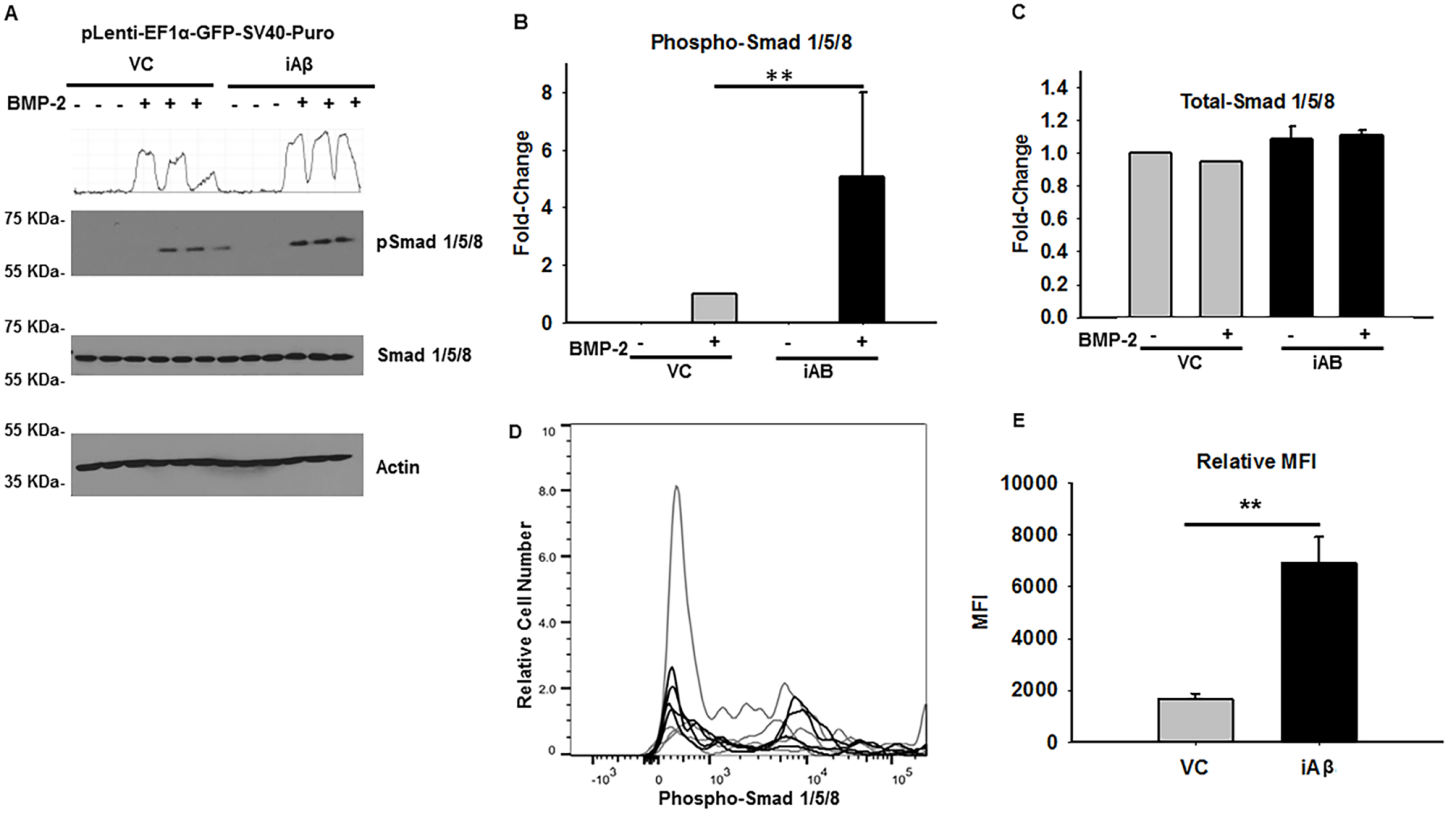
iAβ expression leads to relative increase in BMP-2 dependent Smad 1/5/8 phosphorylation. **(A)** Immunoblot of the phospho-Smad 1/5/8 and total-Smad 1/5/8 in VC or iAβ transduced HEK293 cells following rh-BMP2 stimulation. While total-Smad 1/5/8 levels remain unchanged, an increase in the rh-BMP2 dependent Smad 1/5/8 phosphorylation can be observed in the iAβ transduced cells relative to VC. Two-dimensional gel transformation of phospho-Smad 1/5/8 intensities performed and shown as histogram. Beta-actin used as loading control. **(B)** Plot of the relative densitometry analysis of phospho-Smad 1/5/8 intensity of biological replicates with data shown as mean ± SE (n = 3) with statistical significance (**p < 0.01). **(C)** Plot of total-Smad 1/5/8 abundance depicting unaltered levels among all groups. **(D)** Histogram of phospho-flow cytometry analysis of rh-BMP2 treated VC (gray) or iAβ (black) transduced HEK293 cells. A positive shift in iAβ transduced cells relative to the VC can be seen indicating an increase in the abundance of phospho-Smad 1/5/8. **(E)** Replicate analysis shown as mean ± SE (n = 3) of the MFI with a statistically significant (**p < 0.01) 4.2-fold increase in MFI. Dixon’s outlier analysis was performed on the replicates and there are no outliers within the dataset that should be excluded (p > 0.05).

To orchestrate the complex processes of neurogenesis, differentiation, and maintaining neuronal cell viability, a delicate balance between MAPK and morphogenetic signaling is required. To elucidate how iAβ expression may affect morphogenetic signaling, stably selected VC or iAβ transduced HEK293 cells were treated with vehicle or 100 ng/mL of rh-BMP2 for 1 h, and phosphorylation of Smad 1/5/8 was assessed by immunoblotting (Fig 4A). Densitometry was performed and results were plotted accordingly. Although no change in total-Smad 1/5/8 was observed in either treated or untreated conditions, there was a statistically significant (p < 0.01), 5.06-fold increase in phospho-Smad 1/5/8 upon rh-BMP2 treatment in iAβ expressing cells (Fig 4B and 4C).

Confirmation of these observations was further assessed by phospho-flow analysis wherein an analogous and statistically significant (p < 0.01) 4.2-fold increase in the Smad 1/5/8 phosphorylation was detected (Fig 4D and 4E). In contrast to the iAβ-mediated decrease in MAPK pathway activity, iAβ expression results in an increase in the Smad 1/5/8 mediated morphogenetic signaling cascade. Thus, iAβ expression does not indiscriminately decrease global signal transduction, but rather distinctly alters the intensity and duration of morphogen and MAPK signaling resulting in a potentially pathological dysregulation of the MAPK: morphogen signaling axis.

### Differential influence of extracellular and intracellular amyloid beta on MAPK signaling

**Fig 5:**
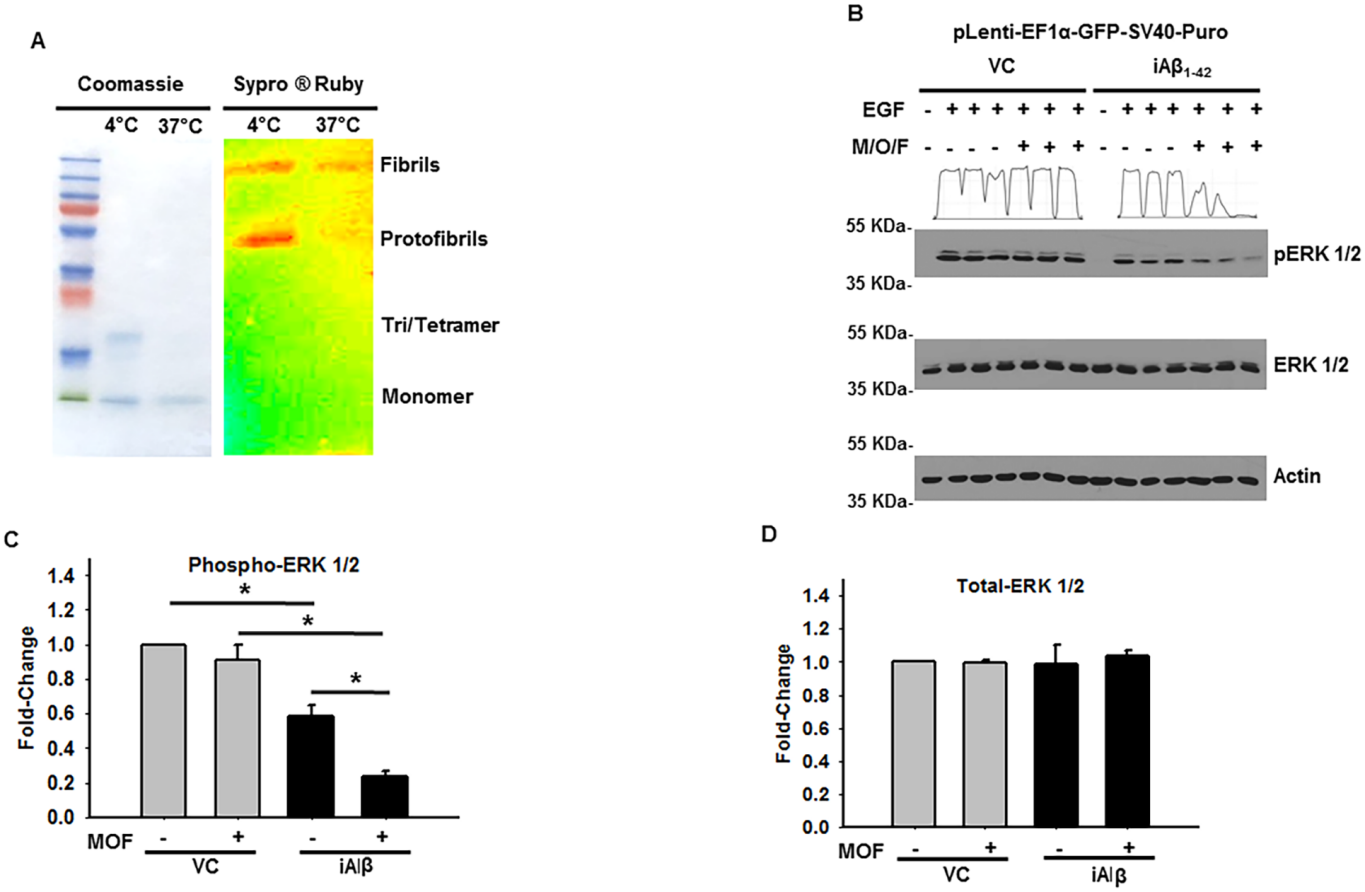
Differential influence of eAβ and iAβ on MAPK activity. **(A)** Confirmation of successful generation of extracellular monomeric, oligomer and fibrillar quaternary organization. Coomassie brilliant blue stained, 15% SDS PAGE gel, showing the varying eAβ quaternary organization states at 4°C and 37°C oligomerization protocols. Preferential staining of lower order eAβ organization observed using coomassie staining at 4°C. Sypro ^®^ Ruby stained, 15% SDS PAGE gel, of eAβ_1–42_ revealing the higher order quaternary organization states. **(B)** Immunoblot of the phospho-ERK 1/2 and total-ERK 1/2 in VC or iAβ transduced HEK293 cells following 24 h pretreatment with monomeric/oligomeric/fibrillary (M/O/F) eAβ and subsequent EGF stimulation. No differences in EGF responsiveness observed in the DMSO or M/O/F treated VC samples. Decreased ERK 1/2 phosphorylation seen in iAβ transduced cells treated with M/O/F relative iAβ transduced cells treated with DMSO alone. Beta-actin employed as loading control. **(C)** Plot of the relative densitometry analysis of phospho-ERK 1/2 intensity in VC or iAβ after treatment with DMSO or M/O/F of biological replicates with data shown as mean ± SE (n = 3) and statistical significance. **(D)** Plot of total-ERK levels demonstrate intensity remains unchanged in all treatment categories. Complete pairwise analysis shown in Table 1.

Given that the pathological features of Aβ_1–42_ are largely attributed to the extracellular activity we have sought to understand the extent to which the extracellular and intracellular localization of Aβ may independently or synergistically affect the MAPK pathway [4]. In addition to its extracellular deposition, the extracellular Aβ_1–42_ (eAβ) undergoes varying degrees of quaternary organization resulting in its oligomerization and fibrillization. Furthermore, monomeric, oligomeric, and fibrillary forms of the eAβ have been proposed to harbor pathophysiological roles in Alzheimer’s disease [25, 26]. Thus, we oligomerized the recombinant amyloid beta and evaluated by 15% SDS PAGE gel stained with coomassie brilliant blue or Sypro ^®^ Ruby. As shown, the staining pattern corresponded with the successful generation of monomeric/oligomeric and fibrillary forms of the eAβ (Fig 5A). Subsequently, a 2 μM cocktail consisting of the pooled monomeric, oligomeric, and fibrillary products (M/O/F) were employed for further analysis of the MAPK signaling activity in the presence and absence of iAβ expression.

Following a 24 h incubation with media supplemented with M/O/F, VC and iAβ transduced HEK293 cells were stimulated with 50 ng/mL of EGF for 1 h before lysates were analyzed by immunoblotting. Although no discernable differences in the total-ERK 1/2 levels were revealed, marked changes in ERK 1/2 phosphorylation were observed among the treatment groups (Fig 5B). Indeed, consistent with our previous observations, a statistically significant (p < 0.05) 1.72-fold decrease in ERK 1/2 phosphorylation was observed in iAβ transduced cells treated with EGF alone. Moreover, while the presence of eAβ had minimal influence on the EGF sensitivity in VC transduced cells, M/O/F pre-treatment of iAβ transduced cells led to a statistically significant (p < 0.05) 3.89-fold decrease in phospho-ERK 1/2 levels. Furthermore, a statistically significant and synergistic 2.63-fold reduction in the ERK 1/2 phosphorylation could be observed between the iAβ untreated and M/O/F treated iAβ samples (Fig 5C and 5D). Complete pairwise analysis shown in Table 1. In combination, this data recommends that Aβ-mediated dysregulation of the MAPK pathway is a molecular consequence distinctly initiated by iAβ. Although eAβ_1–42_ may harbor iAβ_-_independent effects, in this model, the impact of eAβ on MAPK signaling is dependent upon the coincidental expression of iAβ.

### PLX4032 rescues diminished ERK 1/2 phosphorylation

**Fig 6:**
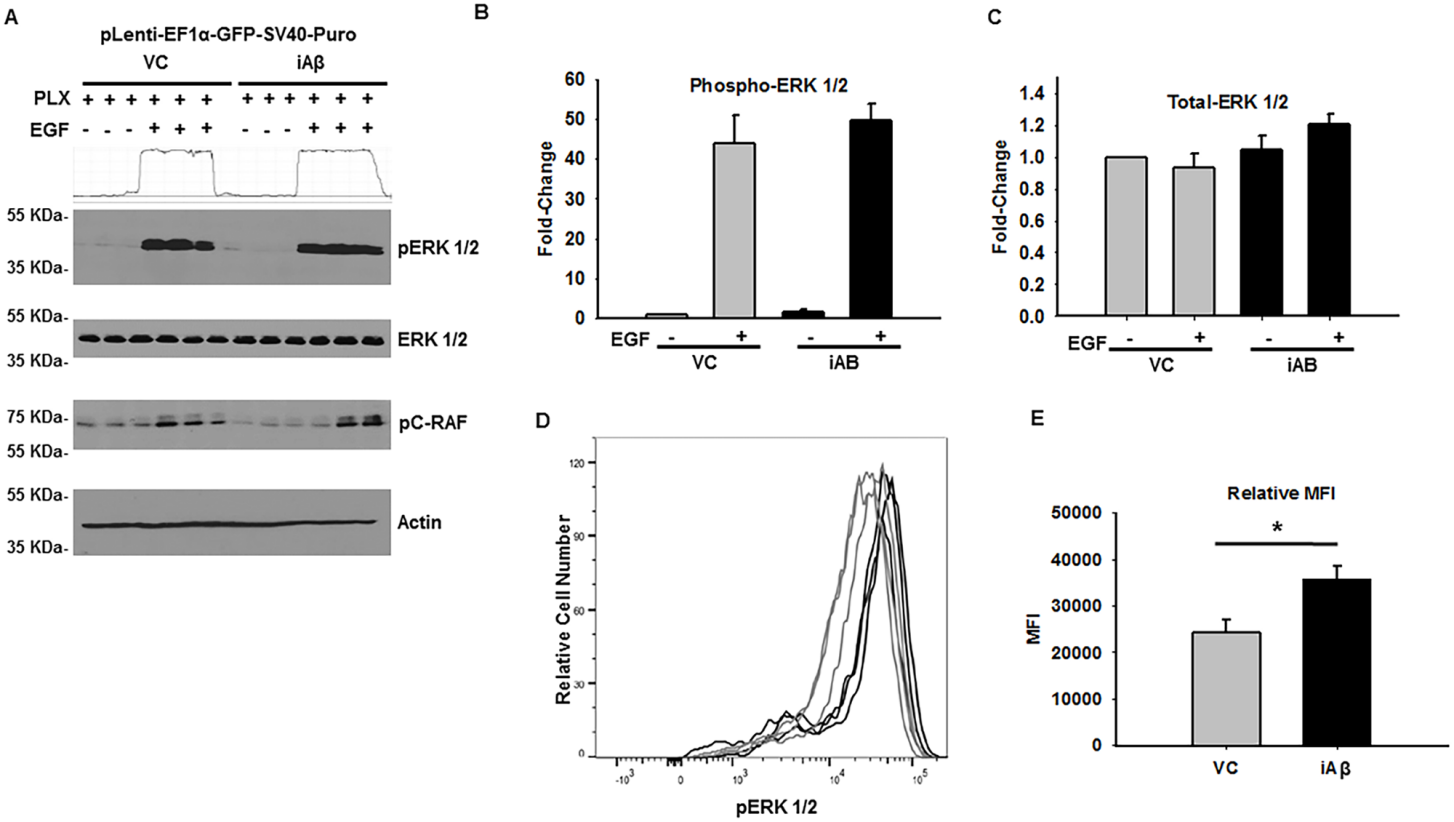
PLX4032 rescue iAβ mediated attenuation of ERK 1/2 phosphorylation. **(A)** Immunoblot for phospho-ERK 1/2, total-ERK 1/2, and phospho-c-RAF (Ser 259) in VC or iAβ transduced HEK293 cells following pretreatment with PLX4032 (30 min) and subsequent vehicle or EGF stimulation. Loss of ERK 1/2 phosphorylation is rescued upon pre-treatment with PLX4032. Two-dimensional gel transformation of phospho-ERK 1/2 intensities performed and shown as histogram. Beta-actin used as loading control. **(B)** Plot of the relative densitometry analysis of phospho-ERK 1/2 intensity of biological replicates with data shown as mean ± SE (n = 3). **(C)** Measurement of total-ERK 1/2 intensity demonstrate unaffected levels following treatment in VC or iAβ transduced cells. **(D)** Histogram of the phospho-flow cytometry measurement of EGF treated VC (gray) or iAβ (black) transduced HEK293 cells for ERK 1/2 phosphorylation. Positive shift in the MFI of iAβ transduced cells reflects an EGF dependent and PLX4032 mediated increase in ERK 1/2 phosphorylation. **(E)** Replicate analysis shown as mean ± SE (n = 3) of the MFI with a statistically significant (*p < 0.05) 1.47-fold increase in MFI following PLX pretreatment and EGF stimulation.

Our examination of iAβ-mediated disruption of MAPK pathway has revealed inhibition of ERK phosphorylation due to reduction in EGFR stability and phosphorylation. Thus, to circumvent the downstream consequences of diminished receptor abundance, it was necessary to promote the MAPK pathway activity in a manner that was independent of receptor concentration. To do this, we employed PLX4032, a small-molecule inhibitor of constitutively active mutant B-Raf^V600E^. However, in the context of wild-type B-Raf, this compound leads to robustly activation of the MAPK pathway [27, 28]. Thus, VC and iAβ transduced HEK293 cells were pre-treated with PLX4032 (0.1 μM) for 30 min prior to incubation with EGF. Lysates were collected and total-ERK 1/2, phospho-ERK 1/2, and phospho-c-RAF were measured by immunoblotting (Fig 6A). While no change in total-ERK 1/2 was detected, both VC and iAβ transduced responded to EGF stimulation. However, in striking contrast to previous observations, no statistical difference (p > 0.05) was observed in PLX4032 pretreated cells stimulated with EGF (Fig 6B and 6C). These findings were further validated by phospho-flow cytometry with a statistically significant (p < 0.05) 1.47-fold increase of phospho-ERK 1/2 in iAβ transduced cells upon pretreatment with PLX4032 (Fig 6D and 6E) Thus, rescue of iAβ-dependent decrease of MAPK signaling could be achieved through PLX4032 activation of downstream effectors of the mitogen signaling cascade.

### *In vitro* maturation and characterization of primary rat cortical and hippocampal neurons

While the transduction of HEK293 cells permitted us to understand the basic functional and mechanistic consequences of iAβ expression, we have further employed cultured primary rat neurons to broaden the significance of these observations in a biologically relevant context. To accomplish this, primary cortical and hippocampal neurons were isolated from embryonic day 18 (E18) rat pups and maintained *in vitro [21]*. To achieve an appropriate neuronal maturation state, the primary cortical and hippocampal cells were grown *in vitro* for 21 days to allow for sufficient neurite growth and synaptic formation prior to any subsequent analysis.

**Fig 7:**
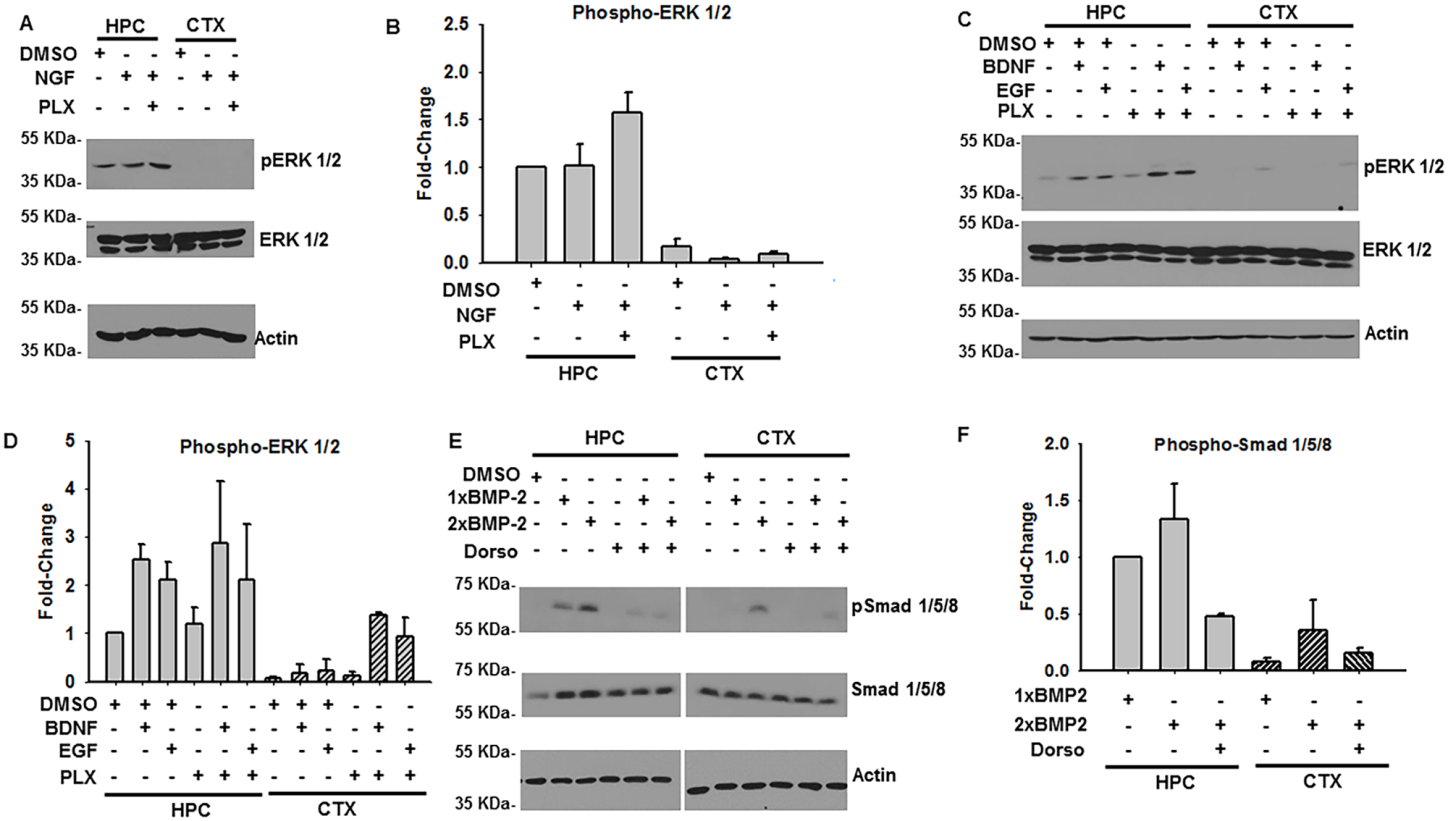
Characterization of primary rat hippocampal (HPC) and cortical (CTX) neuronal sensitivity to growth factor and cytokine. **(A)** Immunoblot of phospho-ERK 1/2 and total-ERK1/2 in DIV21 primary HPC and CTX cells following treatment with DMSO, NGF alone, or PLX4032 pre-treatment and subsequent NGF stimulation. Beta-actin employed as loading control. Although total-ERK 1/2 remains consistent among the treatment groups in both HPC and CTX cells, ERK 1/2 phosphorylation is observed most prominently in HPC cells. **(B)** Plot of the relative phospho-ERK 1/2 abundance relative to DMSO treated HPC cells illustrates the distinct sensitivities of the HPC and CTX populations. Pre-treatment with PLX4032 leads to modest, but statistically significant, increase in NGF dependent ERK 1/2 phosphorylation. Complete pairwise analysis shown in Table 2. **(C)** Immunoblot of phosphorylated and total ERK 1/2 in DIV21 primary HPC and CTX cells following treatment with DMSO, PLX4032, EGF, or BDNF. Beta-actin employed as loading control. Increased HPC sensitivity, relative to CTX cells, can be observed across all treatment combinations. **(D)** Densitometry analysis of relative phospho-ERK 1/2 abundance illustrating the increased HPC to CTX cell sensitivity. Complete pairwise analysis shown in Table 3. **(E)** Immunoblot of phospho-Smad 1/5/8 and total-Smad 1/5/8 of DIV21 primary HPC and CTX cells following treatment with rh-BMP2 alone or in combination with the BMP-2 receptor inhibitor Dorsomorphin. Beta-Actin employed as loading control. Increased hippocampal responsiveness observed relative to cortical samples. **(F)** Plot of densitometry analysis illustrating the increased HPC sensitivity relative to CTX cells. Complete pairwise analysis shown in Table 4.

Before determining how iAβ expression affects MAPK and morphogenetic signaling in cells of neuronal lineage, we first characterized the relative sensitivity of both parental cortical and hippocampal neurons to the growth factor (NGF, EGF, & BDNF) and cytokine panel. Subsequently, primary hippocampal and cortical cells were pre-treated with DMSO or PLX4032, stimulated with NGF, and ERK 1/2 phosphorylation was assessed by immunoblotting (Fig 7A). Although hippocampal cells demonstrated only a marginal increase in phospho-ERK 1/2 in response to NGF alone, a statistically significant (p < 0.05) 1.57-fold increase was observed in cells pre-treated with PLX4032. Furthermore, relative to cortical cells, hippocampal neurons demonstrated a statistically significant (Table 2) increase in responsive to NGF stimulation (Fig 7B).

Similarly, we investigated the relative sensitivity of hippocampal and cortical cells to EGF and BDNF in the presence and absence of PLX4032 pre-treatment (Fig 7C). While treatment of hippocampal cells with either EGF or BDNF led to statistically significant increases in ERK 1/2 phosphorylation, PLX4032 pretreatment did not significantly alter the levels of phospho-ERK 1/2 (Fig 7D). However, examination of the relative responsiveness in hippocampal and cortical cells to these growth factors revealed a statistically significant increase in EGF and BDNF sensitivity in the hippocampal derived neuronal cells (Table 3).

Lastly, we assessed primary hippocampal and cortical neuronal sensitivity to rh-BMP2 (100 ng/mL and 200 ng/mL). While both concentrations of rh-BMP2 were capable of inducing receptor Smad 1/5/8 phosphorylation in hippocampal cells, 200 ng/mL was necessary to elicit a comparable intensity of Smad 1/5/8 phosphorylation in cortical cells (Fig 7E and 7F). Indeed, analysis of cortical and hippocampal responsiveness revealed a statistically significant (p < 0.05) difference in their respective sensitivities to rh-BMP2 (Table 4). When hippocampal and cortical cultures were pre-treated with dorsomorphin, an inhibitor of BMP-2 signaling, Smad 1/5/8 phosphorylation diminished with a substantial trend toward significance (p = 0.051).

Combined, these results have revealed hippocampal cells to be preferentially and significantly more sensitive to the growth factor and cytokine panel. Indeed, the robust response observed upon BDNF, EGF, or rh-BMP2 treatments indicate that this treatment could be similarly employed in our subsequent analysis with transduced cells. Furthermore, consistent with our previous experiments performed with HEK293 cells, pre-treatment of hippocampal cells with PLX4032 did not alter parental sensitivity to the various growth factors employed. Likewise, and to a lesser extent, Dorsomorphin may be used in neuronal cell cultures to induce a comparable decrease in BMP-2 dependent Smad 1/5/8 phosphorylation.

### iAβ expression results in reduced brain-derived neurotrophic factor mediated ERK 1/2 phosphorylation of primary rat hippocampal cells that is rescued by PLX4032

**Fig 8:**
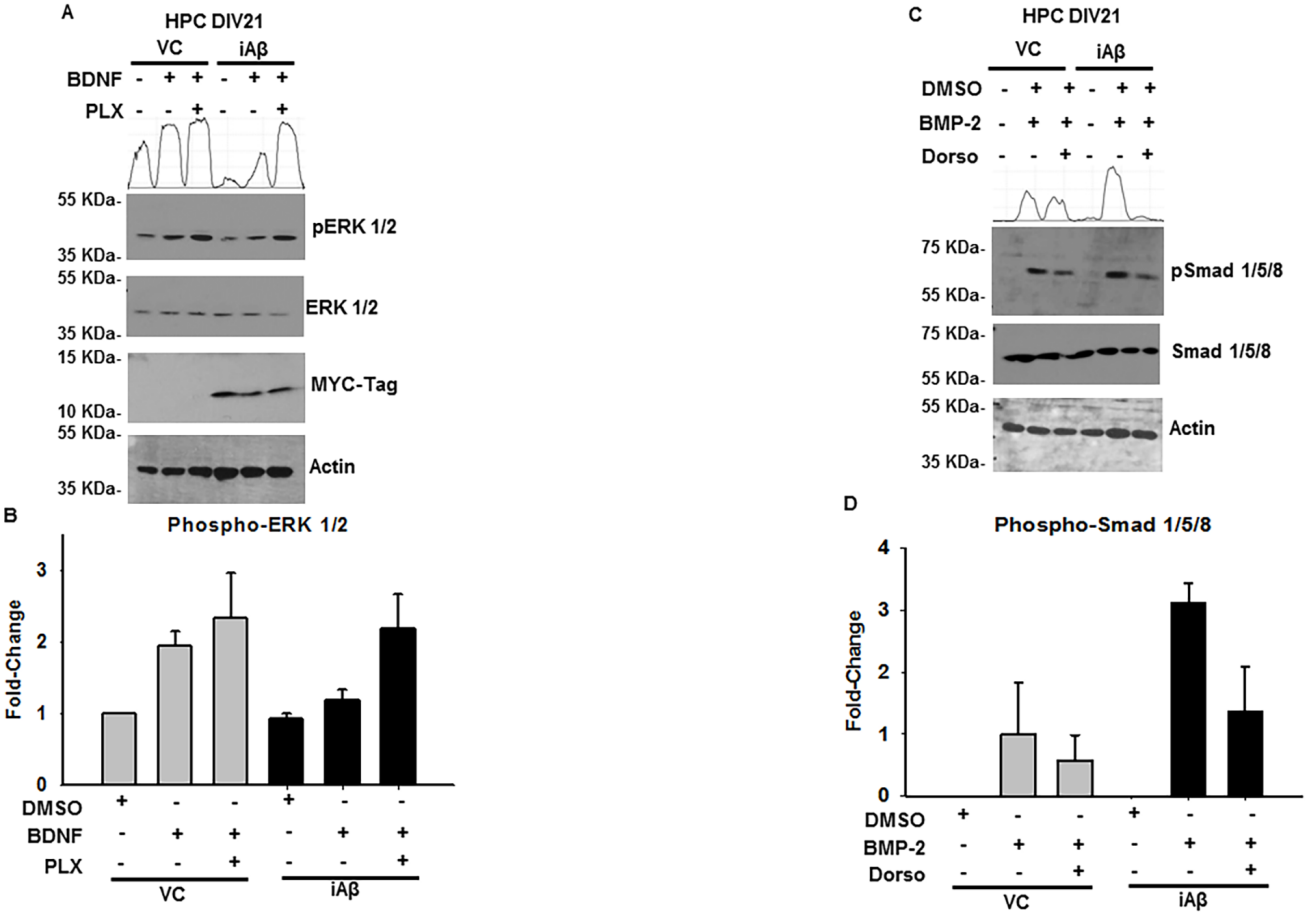
Altered ERK 1/2 and Smad 1/5/8 phosphorylation observed in primary rat hippocampal cells upon iAβ expression. **(A)** Immunoblot of phospho-ERK 1/2, total-ERK 1/2, and MYC-Tag in VC or iAβ transduced HPC neurons following BDNF stimulation. Myc-tag observed in lanes corresponding to iAβ transduced hippocampal cells and absent in VC samples. Decreased ERK 1/2 phosphorylation in iAβ expressing cells is rescued upon pre-treatment with PLX4032. Two-dimensional gel transformation of phospho-ERK 1/2 intensities performed and shown as histogram. Beta-actin used as loading control. **(B)** Plot of relative densitometry analysis of phospho-ERK 1/2 with data corresponding to biological replicates shown as mean ± SE (n = 3). Statistically significant (p < 0.05) decrease in phospho-ERK 1/2 observed in iAβ transduced cells restored upon pre-treatment with PLX4032. Complete pairwise analysis shown in Table 5. **(C)** Immunoblot of phospho-Smad 1/5/8 and total-Smad 1/5/8 in VC or iAβ transduced HPC neurons following rh-BMP2 stimulation. **(D)** Plot of densitometry analysis of biological replicates shown as a mean ± SE (n = 3) depicting an increase in sensitivity, approaching statistical significance (p < 0.0517), of iAβ transduced hippocampal cells relative to VC. Complete pairwise analysis shown in Table 6.

Based upon these findings, we selected BDNF stimulation of cultured hippocampal cells to investigate the impact iAβ may have on MAPK signaling. Accordingly, we transduced cultured hippocampal cells with either the VC or iAβ on DIV17. Next, on DIV21, cells were pretreated with DMSO or PLX4032 (0.1 μM), treated with 50 ng/mL of BDNF, and analyzed by immunoblotting (Fig 8A). Subsequently, we measured phospho-ERK 1/2 intensity and plotted these findings (Fig 8B). As shown, VC transduced hippocampal cells were responsive to BDNF stimulation and exhibited a statistically significant (p < 0.05) 1.95-fold increase in ERK 1/2 phosphorylation. In contrast, iAβ transduced hippocampal cells demonstrated a decrease in BDNF responsiveness and demonstrated a statistically significant (p < 0.05) 1.61-fold reduction in ERK 1/2 phosphorylation. Interestingly, while PLX4032 and BDNF treatment of VC cells did not result in a statistically significant change in ERK 1/2 phosphorylation, treatment of iAβ transduced cells led to a statistically significant increase and restoration of phospho-ERK 1/2 levels comparable to VC intensities (Table 5).

Additionally, we assessed morphogen sensitivity by treatment of DIV21 VC or iAβ transduced hippocampal cells with rh-BMP2 (Fig 8C). Subsequently, phospho-Smad 1/5/8 intensities were measured and plotted (Fig 8D). Although an increase in BMP-2 responsiveness that approached acceptable levels of statistical significance (p = 0.052) was observed (Table 6), further investigation may be necessary before making an explicit conclusion regarding the morphogen sensitivity of hippocampal cells in the context of iAβ expression.

In summary, dysregulated signal transduction, resulting from iAβ expression, demonstrates a molecular and functional conservation in hippocampal cells. Indeed, a significant decrease in BDNF dependent induction of ERK 1/2 phosphorylation was apparent upon iAβ expression and could be rescued by PLX4032. While further investigation is necessary to understand the impact of iAβ expression on the morphogen arm of this axis, disruption of MAPK signaling may be of paramount importance.

## Discussion

As the demographics of our population shift, we are at an ever-increasing likelihood of encountering an age related rise in the incidence of AD [1]. Despite this growing threat, there has been only modest success in improving cognitive function or reducing the neurodegenerative effects of this illness. Although a hallmark of AD, the significance of the Aβ peptide and its fundamental neuropathological mechanism has remained unsettled. However, recent findings have revealed a previously unexplored aspect of Aβ expression, wherein Aβ is not only deposited extracellularly, but is found in numerous intracellular compartments [3]. Furthermore, investigation of EOAD or as a co-morbidity found in patients with Down syndrome has provided further evidence for the early appearance of intracellular accumulation of Aβ that precedes extracellular deposition [6].

The chronology of intracellular and extracellular Aβ expression has prompted further investigation into the pathophysiological consequences associated with peptide localization. Indeed, intraneuronal Aβ has been shown to produce axonal defects with corresponding dysregulation of anterograde and retrograde axonal transport [8]. Consequently, the trafficking of vesicles bearing neurotransmitters, neurotrphins, and endocytosed receptors, critically important to neuronal function, may be negatively affected [29]. Indeed, examination of the effects of intraneuronal and extracellular Aβ has specifically implicated the intraneuronal Aβ species in the dysregulation of hippocampal long-term potentiation and synaptic transmission [10]. Although there is a growing body of research regarding the pathological role of intraneuronal Aβ in synaptic transmission, the impact on signal transduction has not been explored.

As described previously, neuronal cells rely extensively on a balance of neurotrophic and morphogenetic cues to maintain cell viability [30, 31]. Thus, to understand how iAβ may affect this delicate equilibrium, we employed an *in vitro* iAβ lentivirus expression system to investigate its impact on the MAPK and morphogen signaling pathways. Our subsequent investigation has revealed significant alterations of both phospho-EGFR and total-EGFR in EGF treated iAβ-expressing cells. Furthermore, as changes in EGFR abundance were observed only in the context of growth factor stimulation, the differences may be a consequence of decreased post-endocytic receptor stability. Moreover, while many cells may employ variations of endocytic signaling, the unique neuronal morphology has likely made endosomal signaling a necessary adaptation for overcoming the logistical obstacles of transducing signals from axonal/dendritic terminals to the neuronal cell body [12, 32]. Although, we have not investigated this aspect of neuronal signal transduction, it may be an important future consideration as we extend our understanding of iAβ dysregulation of MAPK signaling.

While, the MAPK pathway is a critical positive regulator of cell viability, morphogenetic signaling cues often play an opposing role. Indeed, during neuronal development, the morphogenetic family of signaling molecules are crucial to promoting differentiation and inducing programmed cell death. Furthermore, Smad 1/5/8 phosphorylation is shown to induce neurotrophic addiction in neural precursor cells thereby rendering them more sensitive to reduced neurotrophic support [33, 34]. Upon examination of this signaling modality we have observed a significant upregulation of Smad 1/5/8 phosphorylation in iAβ expressing HEK293 cells. Thus, an increase in morphogen sensitivity combined with the apparent decrease in MAPK signaling may significantly disrupt neuronal homeostasis.

Given the dependence of neuronal cells on neurotrophic support, we subsequently identified strategy that would restore the iAβ-dependent attenuation of MAPK signaling. For this purpose, we have utilized PLX4032 (Vemurafenib) as a novel enhancer of MAPK signaling. Although, this small molecule inhibitor is an effective therapeutic tool used for the treatment of melanoma patients harboring BRAF^V600E^ mutations, a paradoxical activation of the MAPK pathway is observed in a BRAF^WT^ genetic background. Indeed, at low concentrations, PLX4032 induces heterodimerization with CRAF and demonstrates enhanced MAPK signaling upon growth factor stimulation [27]. Subsequently, pre-treatment of iAβ transduced HEK293 cells with PLX4032 led to the restoration of ERK 1/2 phosphorylation intensity similar to or exceeding that of the VC transduced cells.

Although our preliminary characterization of the role of iAβ on MAPK/morphogenetic signaling was performed using HEK293 cells, we further validated our molecular observations using primary rat neuronal cells. Our initial characterization revealed hippocampal cell sensitivity to be disproportionately higher than cortical cells. This cell type specific sensitivity is somewhat mirrored in the regional susceptibility observed in AD, and may account for the particular vulnerability of the hippocampus in AD [35, 36].

**Fig 9:**
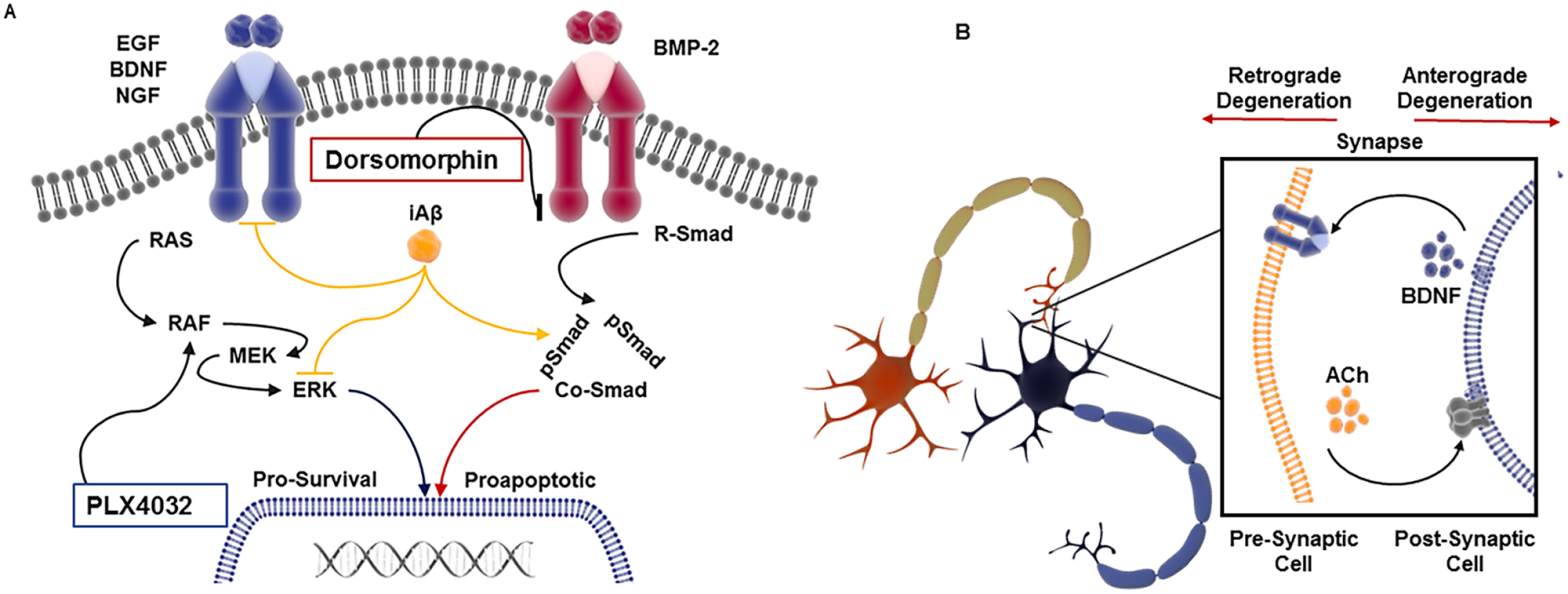
Proposed mechanism of iAβ-mediated neurotoxicity. **(A)** Diagram representative of the molecular dysregulation resulting from iAβ expression. Disruption of the MAPK/morphogenetic axis leads to diminished neurotrophic support resulting from decreased receptor stability that may reduce the intensity of neurotrophic pathways necessary for maintenance of neuronal viability. In contrast, increased sensitivity of the morphogen arm of this axis may further promote this pathological phenomenon. Subsequent downstream rescue using compounds like PLX4032 or inhibition of the morphogen signaling may restore homeostatic signaling intensity. **(B)** Illustration of the cellular influence iAβ may have on pre-synaptic and post-synaptic neurons. Decrease in neurotrophic sensitivity in pre-synaptic neurons resulting from iAβ expression may lead to retrograde neurodegeneration. Consequently, loss of neurotransmitter stimulation from pre-synaptic neurons may lead to post-synaptic anterograde degeneration (Images adapted from Library of Science and Medical Illustrations CC BY-NC-SA 4.0).

On the basis of these observations we transduced cultured primary rat hippocampal neurons on DIV17. Subsequently, cells were pre-treated with DMSO or PLX4032 followed by BDNF stimulation on DIV21. Examination of lysates by immunoblotting revealed a significant decrease in MAPK activation in iAβ transduced hippocampal cells. Strikingly, this decrease in ERK 1/2 phosphorylation was rescued upon pre-treatment with PLX4032. However, when we similarly examined these cells for BMP-2 sensitivity, our finding approached statistical significance, but failed to surpass statistical thresholds. Although pooled cultures of the heterogeneous hippocampal cell population are BMP-2 responsive, the cell sensitivity may not be uniform. Thus, the inherent variability may have influence our ability to assess BMP-2 sensitivity. Although our *in vitro* investigation has led to a number of compelling observations (Fig 9A), this work is limited by certain experimental constraints. *In vivo* validation of these findings will be pursued to further support our conclusions regarding the pathophysiological role of iAβ. However, existing observations made regarding other neuropathies may provide analogous explanations for our findings.

Although the “dying-back” and “dying-forward” phenomenon are models frequently used to describe axonal degeneration observed in peripheral neuropathies, similar mechanisms may be at play in the context of neurodegeneration observed in AD [37, 38]. These models propose a reciprocal relationship between the post-synaptic and pre-synaptic cells whereby a pathological decrease in pre-synaptic or post-synaptic cell function leads to a decrease in the viability of their reciprocal targets. The basis for this relationship resides in the dichotomous contribution made by post-synaptic and pre-synaptic partners. While the pre-synaptic surfaces are structurally and functionally dedicated to the release of neurotransmitters, post-synaptic surfaces are enriched with neurotransmitter receptors necessary for synaptic transmission. In contrast, post-synaptic cells are important producers of growth factors and cytokines that bind to receptor targets on pre-synaptic surfaces. Thus, considered in context of our findings, iAβ-dependent alterations to neurotrophin and cytokine sensitivity may contribute to retrograde degeneration. In contrast, as previously reported, intraneuronal Aβ-mediated decrease in synaptic transmission may contribute to anterograde degeneration of post-synaptic targets [10]. While our investigation has focused on neurotrophin and morphogen sensitivity, the combination of these iAβ-dependent alterations may ultimately contribute to its pathological function. Although we have yet to examine the extent to which iAβ may influence synaptic transmission, it remains an important consideration as we continue to investigate the pathophysiological mechanism iAβ may elicit both retrograde and anterograde neurodegeneration (Fig 9B).

Although we focused on the effects of iAβ expression on MAPK activity, other effects may result from changes to MAPK signaling. In addition to neurodegeneration, cognitive impairment is pathognomonic for AD. While loss of neuronal density is a critical factor, several neurochemical changes occur that impact cognitive decline. In particular, the neurotransmitter acetylcholine (ACh) is deficient in AD patients [39]. Furthermore, NGF stimulation can induce choline acetyltransferase expression and increase the conversion of choline to Ach [40]. Thus, restoration of MAPK activity may affect cognitive function in a similar manner as cholinesterase inhibitors (i.e. Rivastigmine or Donepezil) serve to increase pools of ACh.

In addition to Aβ accumulation, neurofibrillary tangles consisting of hyper-phosphorylated Tau proteins are hallmark developments in AD. Although, a clear pathophysiological relationship between Aβ peptide and Tau phosphorylation has yet to be elucidated, several kinases have been implicated in Tau phosphorylation. In particular, Tau phosphorylation is mediated by glycogen synthase kinase-3 (GSK-3β) [41]. Furthermore, the appearance of intraneuronal Aβ is shown to coincide with GSK-3β activity [9]. While regulation of GSK-3β is complex, growth factor signaling is a central part in maintaining GSK-3β in an inactive state. Thus, in the presence of growth factor stimulation inactive GSK-3β will be prevented from participating in Tau phosphorylation. However, loss of growth factor signaling intensity, as we observed in the context of iAβ expression, may lead to the activation of GSK-3β and subsequent increase in Tau phosphorylation [42].

## Conclusion

Owing to the inherent cellular specialization, neuronal cells may be particularly vulnerable to fluctuations in MAPK/morphogen signaling. As a proof of concept, we have employed an iAβ expression system that has permitted us to model a cryptic yet integral feature associated with the molecular biology of AD. Furthermore, rescue of iAβ_-_mediated dysregulation of MAPK signaling can be partially restored by PLX4032, an enhancer of MAPK pathway (Fig 8B). Although many subtleties regarding this novel shade of Aβ-mediated pathology remain to be investigated, our findings suggest a role for iAβ expression and may also recommend a strategy for targeting the pathological effects of iAβ through restoration of the MAPK/morphogen signaling axis.

## Abbreviations

Ach: acetylcholine
AD: Alzheimer’s Disease
BDNF: brain-derived neurotrophic factor
BMP-2: bone morphogenetic protein 2
DIV: day *in vitro*
eAβ_1–42_: extracellular amyloid beta
EGF: epidermal growth factor
EOAD: early onset Alzheimer’s disease
iAβ_1–42_: intracellular amyloid beta
MFI: median fluorescence intensities
NGF: nerve growth factor
